# Detection and Difference Analysis of the Enzyme Activity of Colloidal Gold Nanoparticles With Negatively Charged Surfaces Prepared by Different Reducing Agents

**DOI:** 10.3389/fchem.2021.812083

**Published:** 2022-01-14

**Authors:** Mingze Ma, Junjun Cao, Ashe Fang, Zhihua Xu, Tieying Zhang, Feng Shi

**Affiliations:** College of Life Science, Shihezi University, Shihezi, China

**Keywords:** colloidal gold, catalyze, nanozymes, Michaelis constant, negative charge

## Abstract

Nanozymes are particles with diameters in the range of 1–100 nm, which has been widely studied due to their biological enzyme-like properties and stability that natural enzymes do not have. In this study, several reducing agents with different structures (catechol (Cc), hydroquinone (Hq), resorcinol (Rs), vitamin C (Vc), pyrogallic acid (Ga), sodium citrate (Sc), sodium malate (Sm), and sodium tartrate (St)) were used to prepare colloidal gold with a negative charge and similar particle size by controlling the temperature and pH. The affinity analysis of the substrate H_2_O_2_ and TMB showed that the order of activities of colloidal gold Nanozymes prepared by different reducing agents was Cc, Hq, Rs, Vc, Ga, Sc, Sm, St. It was also found that the enzyme activity of colloidal gold reduced by benzene rings is higher than that of the colloidal gold enzyme reduced by linear chains. Finally, we discussed the activity of the colloidal gold peroxidase based on the number and position of isomers and functional groups; and demonstrated that the nanozymes activity is affected by the surface activity of colloidal gold, the elimination of hydroxyl radicals and the TMB binding efficiency.

## Introduction

Traditional biocatalysts, including natural enzymes; such as proteins, RNAs, or their complexes, have been shown to be of great value in various biological studies because of their effective catalytic, zymolytic activities under mild conditions (Breaker, 1997; Bornscheuer et al., 2012; Gurung et al., 2013; Zhang et al., 2020). However, natural enzymes are expensive and prone to denaturation due to temperature increase, present of enzyme inhibitors, and humidity increase (Gao et al., 2015). Therefore, it is desirable to find natural enzyme substitutes with strong catalytic activities and stabilities (Kirby, 1994; Yan, 2018). In 2007, Yan et al. discovered for the first time that ferrous oxide magnetic nanoparticles (Fe_3_O_4_ MNPs) possess intrinsic peroxidase-like activity (Gao et al., 2007). The enzymatic properties of nanomaterials were systematically studied from the perspective of enzymology, and corresponding determination standards were established (Jiang et al., 2020). In 2013, Wei et al. defined mimetic enzymes nanomaterials as Nanozymes. Nanozymes are artificial mimetic enzymes with catalytic functions and unique physicochemical properties (Wu et al., 2013). At present, most metal oxides, noble metals, and carbon-based nanomaterials have been found to have enzymatic-like activity, and can simultaneously exhibit the activity of one or more enzymes, such as oxidase, superoxide enzyme, and catalase (Fu et al., 2015; Ray et al., 2017; Yao et al., 2018; Chang et al., 2019; Lin and Wei, 2019). Their catalytic reactions are similar to those of natural enzymes, which not only conforms to the kinetic curve of the Michaelis-Menten equation but also is affected by temperature, substrate concentration, and pH (Zhou et al., 2017).

The catalytic reaction of Nanozymes; is essentially the process of surface electron transfer; the size effects and surface active sites of these materials are key factors that affect the catalytic activity (Peng et al., 2008; Asati et al., 2009; Ahmed et al., 2016; Wu et al., 2019). Kinetic analysis of the enzyme reaction shows that the surface modification of nanoscale biomimetic enzymes can change an enzyme’s affinity for a substrate, thus affecting its catalytic performance (Liu and Liu, 2017; Shi et al., 2019). Therefore, the size and surface modification of Nanozymes have become an important means of regulating the activity of Nanozymes and providing a new route for the further research and application of Nanozymes.

Luo et al. speculated that the reaction followed the Eley-Rideal mechanism (Luo et al., 2010). In addition, JV et al. explored whether colloidal gold has the characteristics of intrinsic peroxidase mimic and applied colloidal gold nanoparticles to glucose detection (Jv et al., 2010). They studied the difference in peroxidase activity between gold nanoparticles modified with different surface charges and unsupported (+)AuNPs; and found through a series of characterization data that the peroxidase activity of the positively-charged AuNPs ((+)AuNPs) was the highest (Jv et al., 2010). Ocsoy et al. have also achieved enhanced catalytic activity of horseradish peroxidase using copper (II) and iron (II) ions (Burcu, 2015; Ocsoy et al., 2015). Hizir et al. reports the multiplexing and adjusting of the intrinsic peroxidase-like activity of gold nanoparticles using DNA and RNA molecules (Hizir et al., 2016).

In this study, reductants with obvious structural differences were used to prepare colloidal gold with similar particle sizes, and enzyme activity was detected. Through the optimization of the morphology and particle size of colloidal gold, the characterization of a series of data, and the detection of enzyme activity, it was found that the enzyme activity of colloidal gold reduced by benzene rings was higher than that of colloidal gold enzyme reduced by linear chains. The above experimental results verified the differences in nanozymes activities with different surface modifications and provide a theoretical basis for the in-depth study and application of nanozymes surface modification.

### Experiment Methods

#### Preparation of Colloidal Gold

To avoid affecting the accuracy of the experimental results, it is necessary to prepare colloidal gold by reduction without any stabilizer. Certain concentrations of the reducing agent, pH regulator and chloroauric acid solution were added in a certain proportion and order, and nanoparticles with similar particle size and morphology were prepared by controlling the reaction conditions (Alkilany et al., 2015). First, 50 ml of water that had been boiled twice was added to a conical flask and heated to the reaction temperature (60–100°C). The reducing agent and HAuCl_4_ solution were added at the same temperature to the conical flask (the order of addition could be adjusted), and the reaction mixture was stirred until the color of the gold nano solution is stable. After preparation, the conical flask was removed and cooled to room temperature, and the original volume was supplemented with water that had been boiled twice to prepare the gold nanosolution. The solution was sealed and stored in a 4°C refrigerator for later use. To maintain the accuracy of experimental results, a control experiment was performed in which stabilizer was not used in the preparation of the gold nanoparticles, due to the nature of the reducing agent. Based on previous studies, the concentrations of the reducing agent (catechol (Cc), hydroquinone (Hq), resorcinol (Rs), vitamin C (Vc), pyrogallic acid (Ga), sodium citrate (Sc), sodium malate (Sm), pH regulator (NaOH, K_2_CO_3_, HCl) and chlorine acid solution were determined based on set proportions, and the components were combined in a specific order. Nanoparticles with similar sizes and morphologies were prepared by controlling the reaction conditions.

The specific proportions are shown in Table 1.

**TABLE 1:** Proportion of reagents for preparing colloidal gold.

Reducing agent concentration	ddH_2_O	HAucl_4_ (1%)	NaOH (0.1 M)	K_2_CO_3_ (0.1 M)	HCl (0.1 M)	Reaction condtions
▲72.7 µL Hq (30 mM)	9.696 ml	■100 µL	★150 µL	-	-	50 °C Stir
▲100 µL Cc (30 mM)	●9.62 ml	■100 µL	-	★100 µL	-	50 °C Stir
▲60 µL R (1%)	●9.74 ml	■100 µL	-	★20 µL	-	Room temperature
▲20 µL Ga (1%)	●9.73 ml	■100 µL	★150 µL	-	-	Room temperature
▲400 µLVc (1.98 mM)	●9.52 ml	■100 µL	-	-	★100 µL	0 °C Stir
▲100 µL St (1%)	●9.72 ml	■100 µL	★100 µL	-	-	50 °C Stir
■100 µL Dm (2%)	●9.8 ml	★100 µL	-	-	-	Boil and stir
■100 µL Sc (2%)	●9.8 ml	★100 µL	-	-	-	Boil and stir

Note(addition order of reagents ● ★ ■ ▲ ◆).

#### Nano-Enzyme Activity Verification

First, the activity of peroxidase-mimicking enzymes was preliminarily verified. It is generally believed that peroxidase-mimicking enzymes can catalyse the decomposition of H_2_O_2_ to produce free radicals that can chemically react with a certain chromogenic substrate. In this study, TMB was used as the chromogenic substrate, and its oxidation product had an absorption peak at 652 nm. The activity of the peroxidase-mimicking enzyme was preliminarily verified according to the change in absorbance. The enzyme activity was measured after confirming that it has peroxidase activity, and a (IU·mg^−1^)was calculated.

The specific proportions are shown in Table 2

**TABLE 2:** Nano-enzyme activity determination verification.

Reactant vial number	NaAc-HAc buffer (mL)	AuNPs(mL)	TMB(µL)	H_2_O_2_(µL)
Vial 1	2	1.2	40	363.7
Vial 2	2.364	1.2	40	0
Vial 3	3.2	0	40	363.7

#### Nanozymes Activity Determination and Catalytic Kinetics Research

Various nanozymes solutions (1.2 ml) were added to vials containing 2 ml of a 0.2 M NaAc-HAc buffer (pH = 3.6), 40 μl of TMB solution (10 mg ml^−1^) was added into the penicillin bottle and mixed. In addition to the above prepared samples, a blank sample was prepared without the addition of H_2_O_2_. The reaction was carried out in the dark at 35°C, and the absorbance at 652 nm was measured every 20 s. The relationship between the absorbance at 652 nm and the reaction time was plotted to obtain the reaction -time curve.

The following formula was used to calculate the activity of the nanozyme (IU·mg^−1^) (Jiang et al., 2018):

$$b_{\mathrm{nanozyme}}=\;V/\left(\varepsilon \times l\right)\times \mathrm{\Delta A}/\mathrm{\Delta t}$$

$$a_{\mathrm{nanozyme}}=b_{\mathrm{nanozyme}}/\left[m\right]$$

By changing the concentration of the substrate H_2_O_2_ within a certain concentration range, the enzyme kinetics of the nanozyme were evaluated by the steady-state kinetic method. TMB was oxidized via the oxygen produced by catalytic decomposition and underwent a chromogenic reaction. As the concentration of the substrate increases, the reaction rate increased linearly and then because saturated at a high concentration. This finding conforms to typical Michaelis-Menten kinetics. The Michaelis-Menten plot was fitted with the substrate concentration and initial catalytic velocity as the horizontal and vertical coordinates, respectively. Then, through the double reciprocal method, the Michaelis equation V=V_max_×[S]/(K_m_+[S]) was used to calculate the reaction kinetics parameters. In this equation Km is the Michaelis constant, V is the initial rate of the reaction, and [S] is the concentration of the substrate. The Michaelis constant is a measure of the ability of the enzyme to bind to the substrate. The smaller the Km value is, the stronger the binding capacity to the substrate, the greater the Km value and the weaker the affinity.

## Results and Discussion

### Optimization of Nanoparticles

Due to the requirements of experimental research, spherical gold nanoparticles of identical size were synthesized. The characterization outcomes demonstrated that the colloidal gold, produced via the methods in the relevant literature (Turkevich et al., 1954; Lee and Park, 2011; Balbuena Ortega et al., 2014; Luo et al., 2019; Liu et al., 2017), has different sizes and various morphologies, except for some linear chain organic acids (tartaric acid, malic acid, citric acid) and catechol. Resorcinol cannot undergo rapid oxidative self-conversion into its quinone forms because of the lack of electronic resonance in the aromatic nucleus; therefore, its slow reduction, unlike that of other hydroxylphenols, causes colloidal gold to exhibit irregular diameters. The negative and positive charges of hydroxylphenols, whose magnitudes also affect the ability to gain and lose electrons, are mainly distributed on the oxygen and the aromatic ring. Compared with other hydroquinones, 1,4-hydroquinone has the strongest electron gain and loss ability. Colloidal gold fabricated using pyrogallic acid to reduce the corresponding metal ions (Au^3+^) is affected by the quantity and position of their hydroxyphenols, which makes their charge distribution uneven and ultimately leads to the uneven particle size. The formation of colloidal golds mediated by ascorbic acid occurs through the release of protons and electrons in an acidic medium, and the freed electrons eventually reduce Au (Ⅲ) ions into colloidal gold particles, which are then covered by dehydroascorbic acid (DHAA) (Rastogi et al., 2017). The four alcoholic hydroxyl groups contained in nanoparticles are extremely reductive, as observed from the structure of ascorbic acid, which ultimately results in the inhomogeneity of the colloidal gold sizes caused by the rapid reaction. In summary, there are two feasible main options for acquiring colloidal gold nanoparticles with uniform particle size and a spherical shape. The first is to add a stabilizer during the preparation process, and the second is to adjust the temperature and pH changing the reducing agent and reaction rate. We eventually chose the second option because it was realistically more feasible. The results shown in Table 1 are the specific optimization methods.

### Characterization of Nanozymes

**FIGURE 1:**
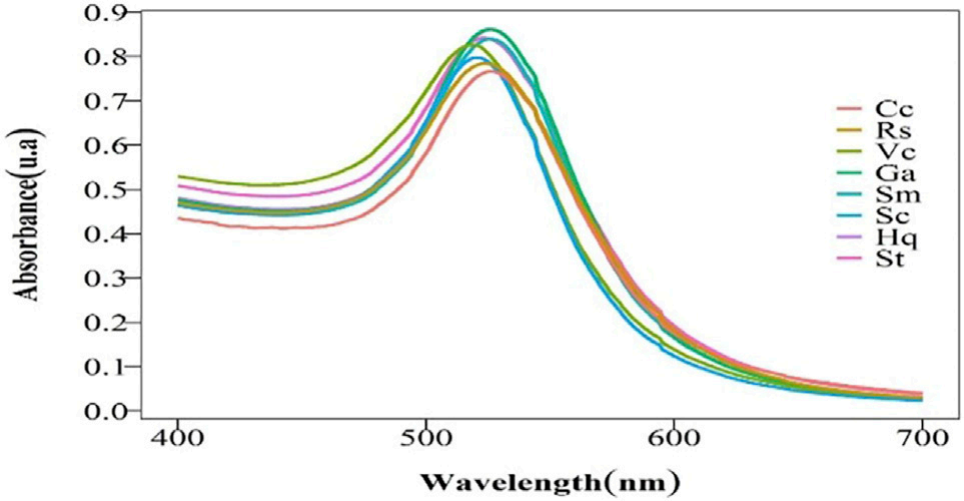
Visible light (400–700 nm) absorption spectra of colloidal gold (λ/nm).

It is generally accepted that both the temperature and pH or enzyme concentration have a strong influence on enzyme activity. On the basis of the experiment carried out at room temperature, at a pH of 3.6, the OD values of all kinds of colloidal gold are similar according to UV-vis spectroscopy (Figure 1). The results showed that the concentrations of colloidal gold were extremely similar, which suggests that enzyme concentration dose not have a strong impact.

**FIGURE 2:**
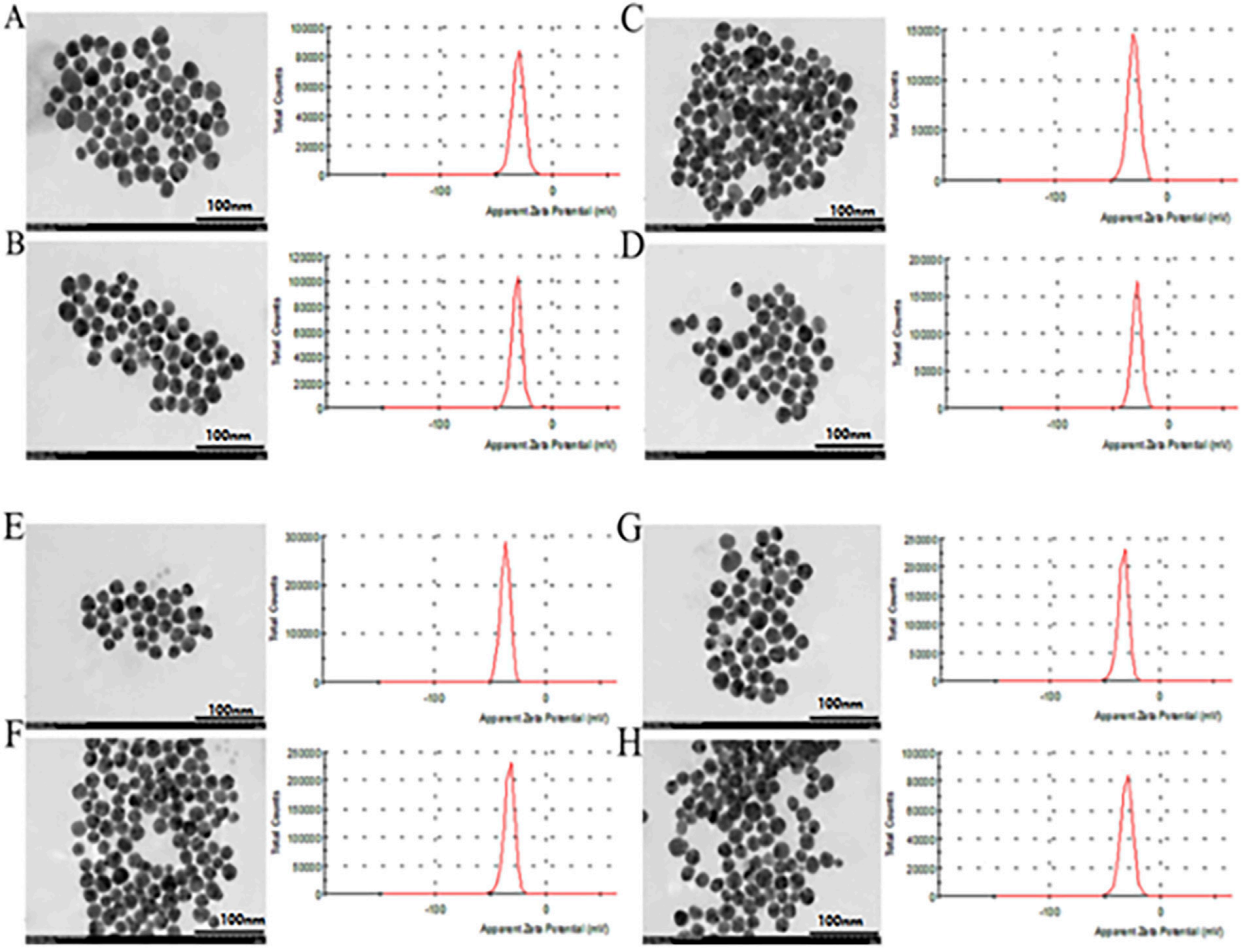
The particle size distribution, TEM and zeta potential of colloidal gold prepared by reduction with Cc **(A)**, Hq **(B)**, Rs **(C)**, Vc **(D)**, Ga **(E)**, Sc **(F)**, Sm **(G)** and St **(H)**.

However, the size and surface modification of colloidal gold significantly impact the activity of the nanomaterials, illustrating that it is vital to fabricate colloidal gold with similar particle sizes. As shown in Table 1, the concentration and doses of various reducing agents are different because the agents have large structural differences. It can be concluded from the maximum UV-vis absorption peak that there is little difference in the sizes of the nanoparticles prepared by different reducing agents (Figure 1). Transmission electron microscopy (TEM) images also reflect this result (Figure 2) (Average particle size of different colloidal gold: Cc (A) 21.2 ± 6.5nm, Hq (B) 21.4 ± 6.6nm, Rs (C) 22.7 ± 7.4nm, Vc (D) 22.5 ± 4.9nm, Ga (E) 21.9 ± 4.9nm, Sc (F) 20.6 ± 6.3nm, Sm (G) 22.6 ± 4.9nm, St (H) 20.8 ± 6.5 nm)

The zeta potential shows that the surface charges of all the colloidal gold particles are negative. (Figure 2).

### Nanozyme Activity Verification

**FIGURE 3:**
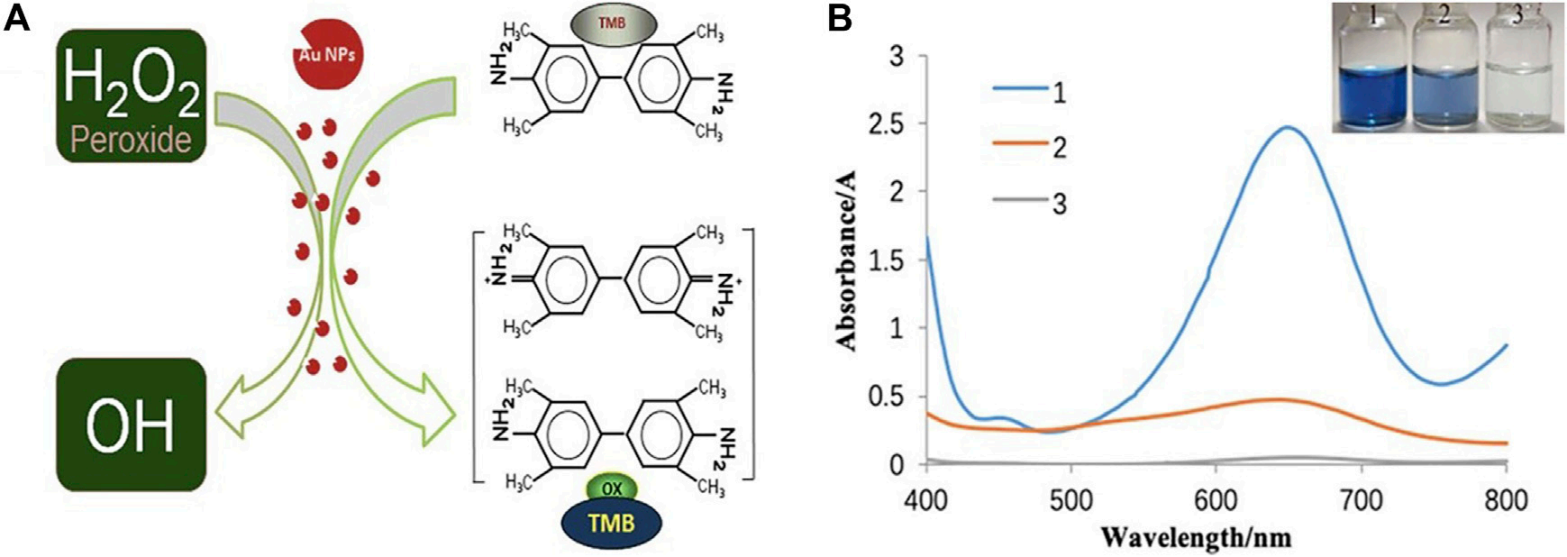
**(A)** Mechanism of peroxidase activity of colloid gold **(B)** UV spectrogram and photo of nanozymes activity verification. (1: AuNPs at TMB at H_2_O_2_; 2: AuNPs at TMB; 3: TMB at H_2_O_2_).

The activity of the biomimetic enzyme was initially verified by the development of colour during the reaction, and the catalytic ability of the enzyme was determined, as shown in Figure 3.

The activities of eight prepared nanozymes indicated that their catalytic effects were similar. When hydroquinone was used as the reducing agent, for example, as shown in Figure 3B, the clear solution in bottle #1, which contained both nanozymes, TMB and H_2_O_2_, gradually turned blue, and the product had a strong absorption peak at 652 nm, which was attributed to TMB oxidation. This oxidation occurred because H_2_O_2_ decomposed after being catalysed by nanozymes to produce a large amount of O^2-^ in the system, this ion oxidized TMB causing the colour to change to blue (Figure 3A). A slightly blue colour appeared in the solution of the second bottle containing Nanozymes and TMB, and the product also had a faint peak at 652 nm which was judged to be the absorption peak of oxdized TMB, which was oxidized to a lesser degree by a small quantity of oxygen-containing substances adsorbed on the surface of the nanozymes. Because the contents of bottle #3, which contained TMB and H_2_O_2_, did not change colour, it was concluded that H_2_O_2_ did not decompose, and TMB was not oxidized when the nanozymes were added to the system. In conclusion, nanozymes possess catalytic peroxidase activity. To determine the unambiguous differences between Nanozymes fabricated by various reducing agents, the enzyme activity and enzymatic reaction kinetics were explored.

Study on activity determination and catalytic kinetics of nanozymes.

#### Research on the Most Suitable Conditions for Nanozymes Activity

**FIGURE 4:**
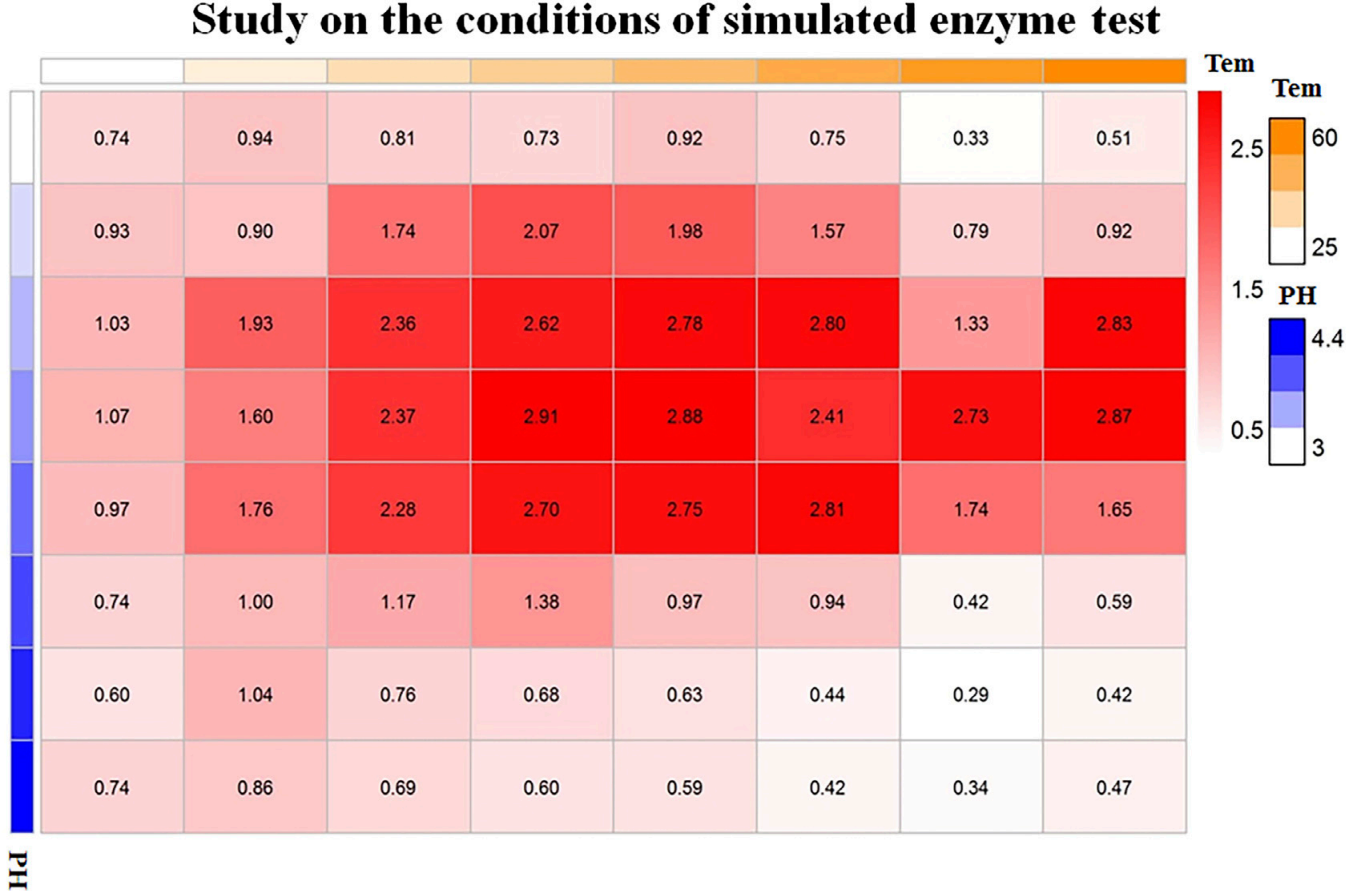
Orthogonal analysis of the pH and temperature of colloidal gold prepared by sodium citrate reduction.

Taking the preparation of colloidal gold by Sc reduction as an example, through orthogonal design under different temperature and pH conditions, the optimal temperature and pH conditions were determined, as shown in Figure 4. When the pH was 3.6 and the temperature was 40°C, the OD value (2.91) is the highest, which reflects the highest activity of the nanozymes.

#### Determination of Nanozymes Activity

**FIGURE 5:**
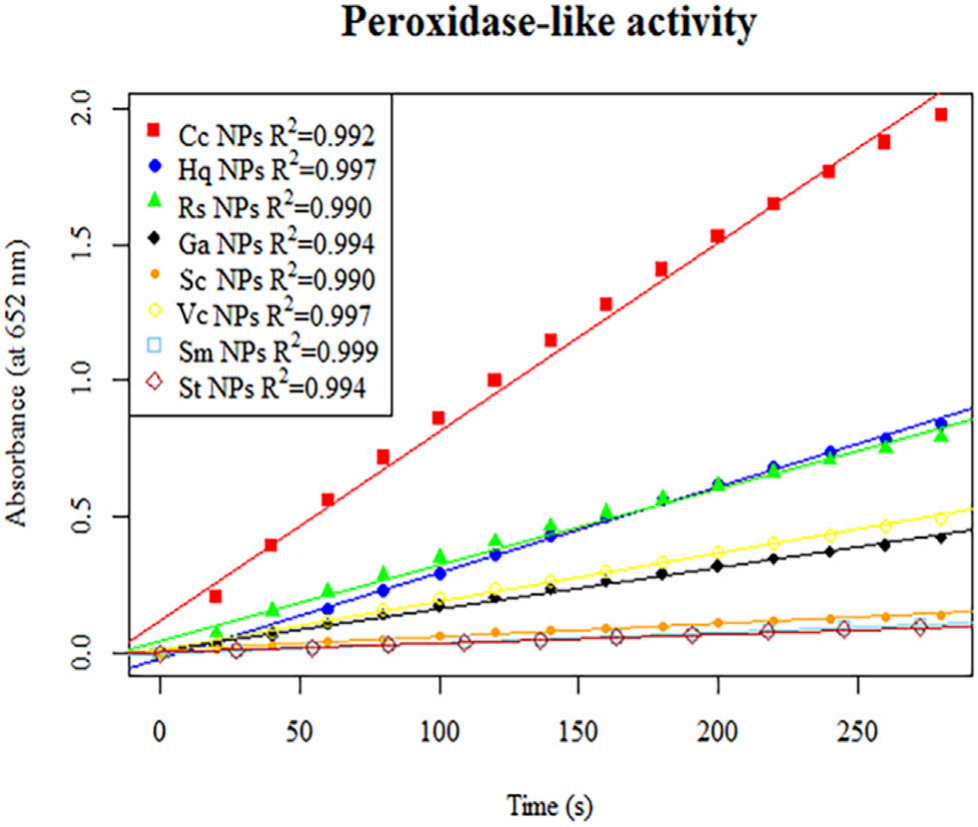
The initial linear section of the reaction–time curves of the colorimetric TMB reaction catalysed by colloidal gold nanozymes prepared with eight different reducing agents, Cc, Hq, Rs, Vc, Ga, Sc, Sm and St.

**FIGURE 6:**
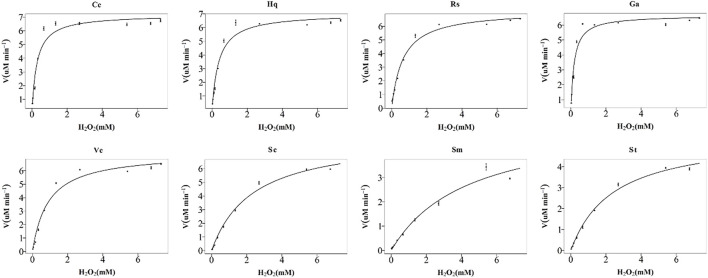
Michaelis–Menten plots for nanozymes prepared with eight different reducing agents, Cc, Hq, Rs, Vc, Ga, Sc, Sm, and St. The concentration of TMB used was 1%, and the H_2_O_2_ concentration varied from 0 to nearly 30%. The error bars represent the s. e. derived from three independent experiments.

The peroxidase-like activity of colloidal gold nanoparticles results from their own activity. Before use, the prepared colloidal gold was centrifuged and then redispersed in ultrapure water to confirm the accuracy of the experiment and eliminate the influence of other impurities. H_2_O_2_ was decomposed into two HO^−^ radicals, which adsorbed on the surface of the AuNPs and were stabilized by the AuNPs through the interaction of partial electron exchange (Perez-Benito, 2004). Finally, the catalytic performance of the AuNPs was improved. In this experiment, the effects of reductants with different chemical structures on the properties of the prepared biomimetic enzymes were explored by investigating the enzyme activities and catalytic reaction kinetics of the mimic enzymes. According to the standard method provided by Yan (Jiang et al., 2018), the initial reaction rate under the condition of substrate saturation was selected as the standard to evaluate the enzyme activity. The rate of change in absorbance over time was converted into that of concentration over time according to the Beer-Lambert Law. The activities of these eight enzymes were in the following sequence from high to low, Cc, Hq, Rs, Vc, Ga, Sc, Sm and St (Figure 5). The control variable method was used to analyse whether the difference in colloidal gold surface modification leads to the difference in the affinity for the substrate. The affinity of the enzyme for the two substrates, H_2_O_2_ and TMB, was compared. When the substrate was H_2_O_2_, the Km values were in the order of Sm, St, Sc, Vc, Rs, Hq, Cc and Ga (Figure 6). The affinity of the biomimetic enzyme for the substrate had an almost a linearly positive correlation with the reducibility of the reductant, and the stronger the reducibility of the simulated enzyme was, the greater the affinity of the biomimetic enzyme for the substrate. Table 3 shows that the affinity of the mimic enzyme modified by benzene ring is strongest, and the affinity of the biomimetic enzyme modified by Vc was stronger than that of the biomimetic enzyme modified by the linear chain. The surface properties of the AuNPs changed as well. Due to the absorption of H_2_O_2_, the electron transfer processes mediated by particles are different. The stronger the reducibility of the reductants is, the easier it is for the reductants to bind to the colloidal gold surface and the easier it is for charge transfer occur, making the colloidal gold surface more active and facilitating H_2_O_2_ adsorption on the colloidal gold surface to form HO^−^ radicals (Cui et al., 2005). when TMB was used as the substrate, the Km value magnitudes, when the substrate is TMB are in the following order: Hq, Rs, Cc, Ga, Vc, Sc, St, Sm (Table 4). Research shows that the activity of colloidal gold peroxidase was positively correlated with the affinity of the substrate hydrogen peroxide (Zhang et al., 2020). As shown in Table 5, the colloidal gold peroxidase activity modified by eight different reducing agents is in order Cc, Hq, Rs, Vc, Ga, Sc, Sm, and St (from high to low).

**TABLE 3:** Km and Vmax with H_2_O_2_ as substrate.

Reducing agent	Cc	Hq	Rs	Ga	Vc	Sc	Sm	St
Km (mg/ml)	0.2489	0.4971	0.3782	0.2258	0.8490	1.634	2.752	2.382
V_max_	6.596	6.057	6.12	5.385	6.897	4.228	3.785	6.369

Note: K_m_ is the Michaelis constant, V_max_ is the maximum reaction velocity.

**TABLE 4:** K_m_ and V_max_ with TMB as substrate.

Reducing agent	Cc	Hq	Rs	Vc	Ga	Sc	Sm	St
Km (mg/ml)	0.0074	0.0153	0.0105	0.003	0.0059	0.0026	0.0008	0.0016
V_max_	6.689	4.496	5.010	6.039	3.495	1.866	1.306	2.729

Note: Km is the Michaelis constant, V_max_ is the maximum reaction velocity.

**TABLE 5:** The results of enzyme activity measurements.

Reducing agent	Cc	Hq	Rs	Vc	Ga	Sc	Sm	St
Amount nanozyme added	4 ml							
	500 µL							
Nanozyme concentration (mg/ml)	0.0816	0.1	0.1	0.1	0.1	0.1	0.1	0.1
b _nanozyme_ (IU)	0.058	0.023	0.018	0.012	0.011	0.004	0.0023	0.0022
a _nanozyme_ (IU·mg^−1^)	1.415	0.464	0.378	0.242	0.219	0.084	0.047	0.044

Note: a _nanozyme_ and b _nanozyme_ are calculated from the formula. (Page 4, Line 97,98).

**FIGURE 7:**
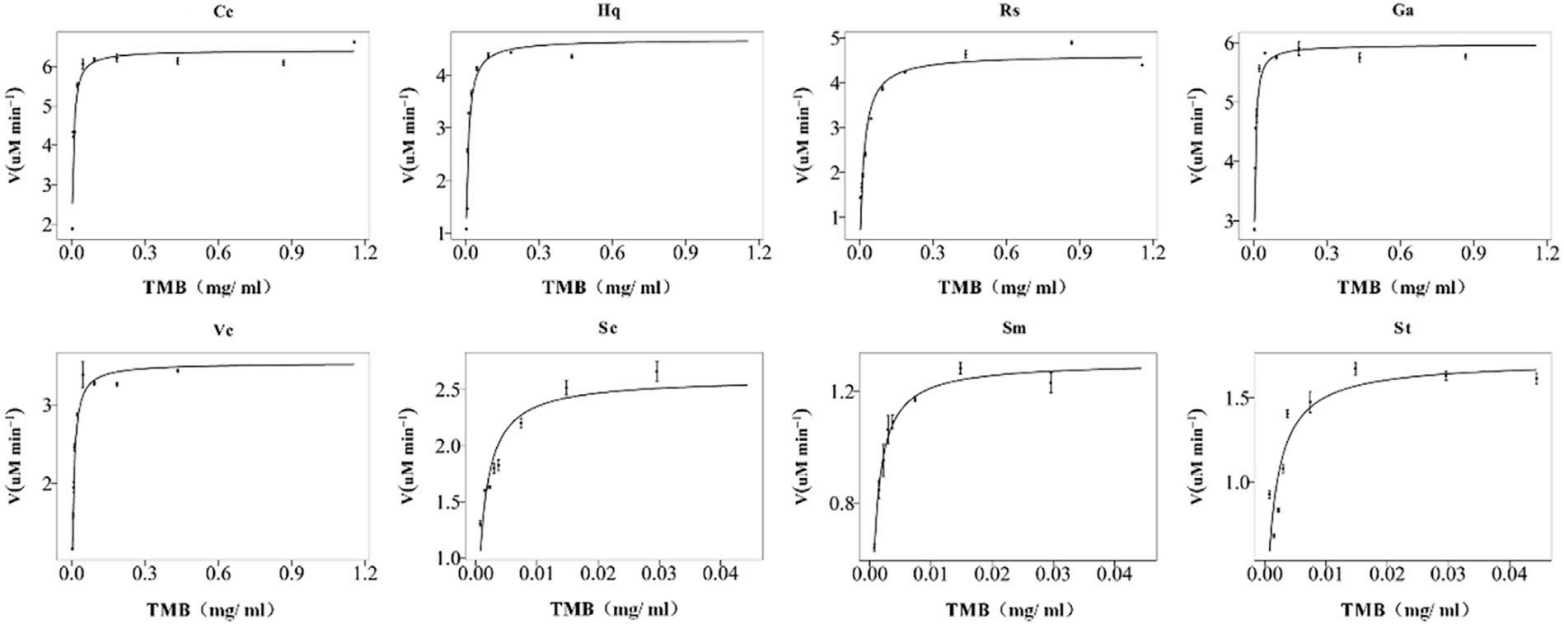
Michaelis–Menten plots of the nanozymes prepared with eight different reducing agents, Cc, Hq, Rs, Vc, Ga, Sc, Sm, and St. The concentration of H_2_O_2_ used was 30%, and the TMB concentration varied from 0 to nearly 1%. The error bars represent the s. e. derived from three independent experiments.

The reasons for the differences were taken into consideration (Figure 7). First, due to the resonance between the nonbonding PZ orbital of the phenolic hydroxyl oxygen atom and the π-bonding orbital of the benzene ring, the phenolic hydroxyl oxygen has a higher electron density (Li et al., 2011), and it was bound to the surface of colloidal gold, which greatly increased its reactivity. Second, as a radical scavenger, Vc competed with TMB, which resulted in the incomplete reaction between the HO^−^ radicals and TMB. Finally, the colloidal gold nanoparticles capped with carboxyl groups (citrate, etc.) tended to electrostatically attract the amino groups of TMB, moreover, the carboxyl moieties of linear chain organic acids are more easily adsorbed on the surface of colloidal gold, resulting in relatively fewer exposed colloidal gold surface moieties, and leading to the relative difficulty of H_2_O_2_ adsorption on the colloidal gold surface. Compared with nanoparticles modified with other structures, their affinity for TMB was greater. The results showed that the affinity of the nanozymes prepared by the reduction of phenolic compounds was higher than that of the nanozymes reduced by linear chain organic acids.

**FIGURE 8:**
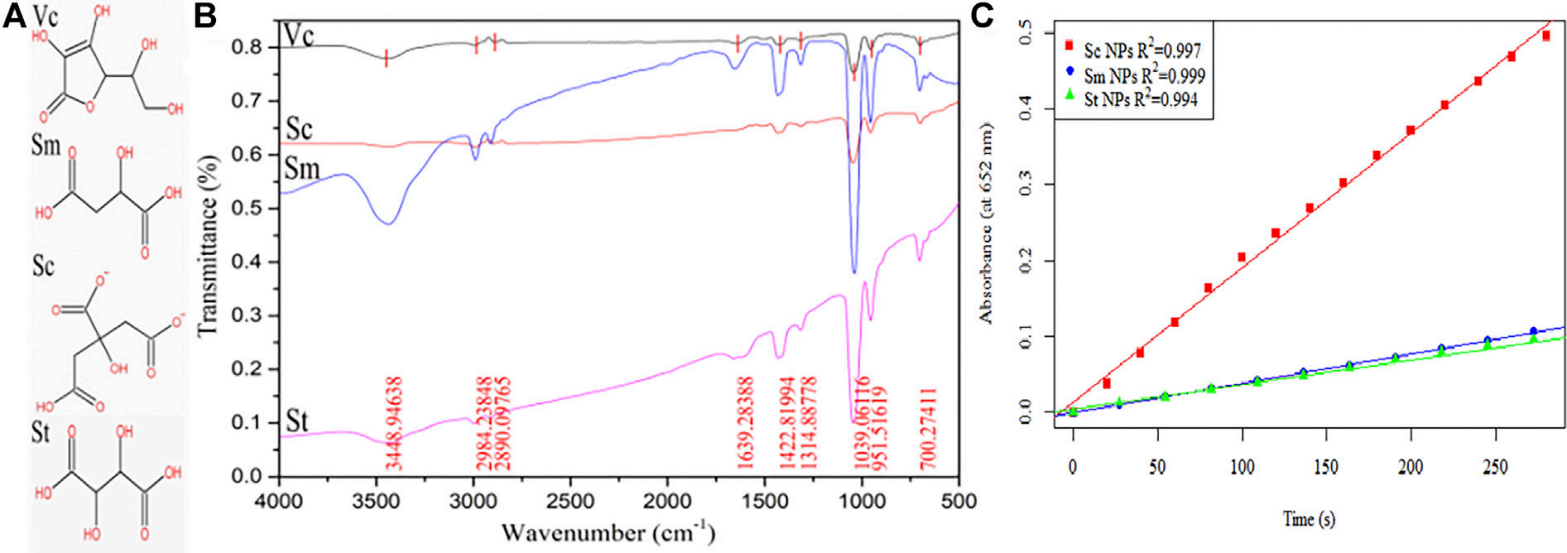
**(A)** The molecular structure of Vc, Sc, Sm and St and **(B)** the infrared spectra of the prepared colloidal gold nanoparticles after reduction. **(C)** Part of Figure 5.

The IR spectra of the colloidal gold prepared by Vc, Sc, Sm and St reduction were analysed (Figure 8B), and the results showed that there was a -OH stretching vibration peak at 3,449 cm^−1^. The results indicate that the peak intensity of Sc is the lowest, but the peak intensity of Sm is very strong because it may contain water molecules. The wavenumbers 2984 cm^−1^ and 2,980 cm^−1^ are due to C-H stretching vibrations on saturated carbon chain, those at 1750–1,680 cm^−1^ are carbonyl stretching vibration peaks, and those at 1,422, 1314, and 1,039 cm^−1^mainly occur due to C-H in-plane bending vibrations and single C-C skeleton vibration. However, during the colloidal gold preparation process, the ring of Vc is opened and the double bond is reduced, resulting in the disappearance of two characteristic IR peaks. To further study the effect of the amount and position of carboxyl and hydroxyl groups in linear chain organic acids on the enzyme activity, three linear chain organic acids with insignificant structural differences (tartrate, citrate, malate) were used as reductants to prepare colloidal gold, and the enzymatic-like activity of colloidal gold was examined. The detection results are shown in Figure 8C, and the enzymatic activities were in the order of sodium citrate > sodium malate > sodium tartrate. Citric acid is tricarboxylic acid, while malic acid and tartaric acid are dicarboxylic acids. Malic acid has one fewer alcohol hydroxyl group than tartaric acid. Under conditions with the same carbon skeleton, tartaric acid has one more hydroxyl group than malic acid, which eventually leads to the easy elimination of hydroxyl radicals generated from hydrogen peroxide adsorbed on the surface of colloidal gold. Malic acid has one fewer carboxyl group than citric acid. Although they both contain hydroxyl groups, due to bond angle differences, the hydroxyl groups of citric acid are less active than those of malic acid which ultimately leads to the generation of hydroxyl radicals that better bind to TMB.

**FIGURE 9:**
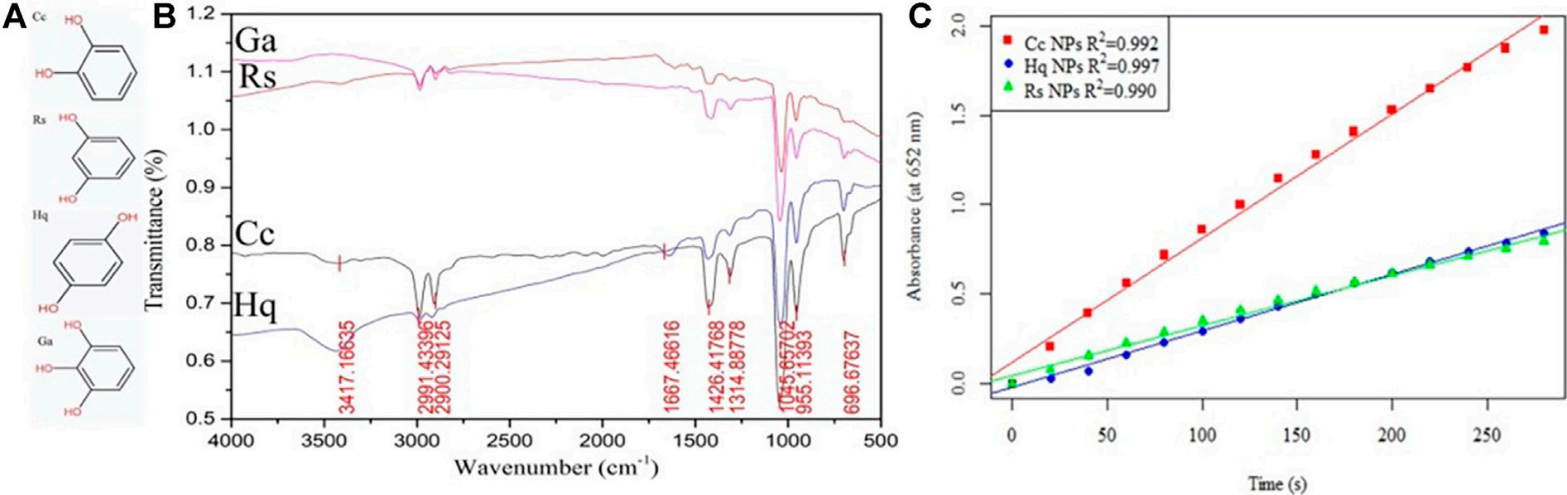
**(A)** The molecular structure of Cc, Hq, Rs, Ga and **(B)** the infrared spectrum of the reduced colloidal gold **(C)** Part of Figure 5.

The colloidal gold prepared by four kinds of phenol reduction method were analysed by FTIR (Figure 9B). There is a broad peak at 3,417 cm^−1^, which is attributed to the stretching vibration of water molecules. The C-H stretching vibration peaks appear at 2,991 cm^−1^ and 2,900 cm^−1^, the quinone carbonyl stretching vibration peak appears at 1,667 cm^−1^ and benzene absorption peaks appear at 1,426 cm^−1^ and 1,314 cm^−1^. However, no characteristic peaks of four phenols were found in the fingerprint area, indicating that all phenols participated in the reduction and stabilization of colloidal gold. Experimental studies on Cc, Rs, and Hq were carried out mainly to comparatively analyse the differences in the nanozyme activity of prepared colloidal gold from the perspective of isomers. Due to their different molecular structures, the ease of their redox reactions varies. The hydroxyl group on the benzene ring belongs to electron withdrawing group, but because its conjugation effect is greater than its electron withdrawing ability, the hydroxyl group will eventually act as electron donating group. The two hydroxyl groups on the phenyl ring differ in position and therefore in the distribution of charge density, which leads to differences in their degree of redox. Among the three isomers, when the two hydroxyl positions are in the para position of benzene ring, the charge density is the highest, followed by the ortho position and the meta position. However, because the phenolic hydroxyl group of catechol is in the ortho position, their electron cloud densities influence each other, which greatly enhances the charge effect. Finally, the colloidal gold prepared by catechol reduction method had the highest surface activity, followed by hydroquinone and resorcinol.

## Conclusion

Colloidal gold nanoparticles with similar particle sizes were prepared by optimized processes, and the enzyme activities of eight different surface-modified colloidal gold samples were verified. The results showed that the enzymatic-like activity of the colloidal gold reduced by benzene ring is higher than that of colloidal gold enzyme reduced by linear chain. The effects of isomers, and the number and location of functional groups on the enzymatic-like activity of colloidal gold prepared with three different hydroquinones and three kinds of linear chain organic acids as reductants were discussed separately. Hydroquinone affected the reactivity of the colloidal gold surface, resulting in a different number of hydroxyl radicals produced, while linear chain organic acids affect the reaction efficiency of hydroxyl radicals and TMB, resulting in a difference in enzyme activity.

## Data Availability

The original contributions presented in the study are included in the article/Supplementary Material, further inquiries can be directed to the corresponding author.
